# Serum Triamcinolone Levels following Cervical Interlaminar Epidural Injection

**DOI:** 10.1155/2018/8474127

**Published:** 2018-03-21

**Authors:** Tim J. Lamer, Rozalin R. Dickson, Halena M. Gazelka, Wayne T. Nicholson, Joel M. Reid, Susan M. Moeschler, W. Michael Hooten

**Affiliations:** ^1^Department of Anesthesiology, Mayo Clinic College of Medicine, Rochester, MN, USA; ^2^Division of Pain Medicine, Mayo Clinic College of Medicine, Rochester, MN, USA; ^3^Division of Medical Oncology, Mayo Clinic College of Medicine, Rochester, MN, USA; ^4^Division of Molecular Pharmacology and Experimental Therapeutics, Mayo Clinic College of Medicine, Rochester, MN, USA

## Abstract

**Background:**

Cervical interlaminar epidural steroid injections (ESIs) are commonly performed procedures to treat painful cervical radiculopathy, but little is known about the systemic absorption and serum levels of steroids following injection. The primary objective of this study was to investigate the pharmacokinetics of fluoroscopy-guided cervical epidural-administered triamcinolone acetonide in a cohort of patients with cervical radicular pain seeking treatment in a pain medicine clinic.

**Methods:**

The study cohort included eight patients undergoing a fluoroscopically guided C7-T1 interlaminar ESI at a pain medicine specialty clinic. Blood was collected prior to the ESI and on days 1, 2, 4, 6, 8, 14, 21, 28, 35, and 42 following the injection. The sample extract was analyzed by tandem mass spectrometry.

**Results:**

The terminal elimination half-life of cervical epidural-administered triamcinolone in a noncompartmental analysis was 219 hours. In the noncompartmental analysis, peak triamcinolone concentrations of 5.4 ng/mL were detected within 22.1 hours after administration.

**Conclusions:**

The pharmacokinetics of cervical epidural-administered triamcinolone is consistent with our previous study of lumbar ESI, demonstrating that the elimination half-life is longer than that which has been reported following intravenous triamcinolone. The elimination half-life was shorter following cervical ESI than that which has been reported following lumbar ESI.

## 1. Introduction

Cervical interlaminar epidural steroid injection (ESI) is a commonly performed procedure in pain medicine used to treat painful cervical radicular pain syndromes [1]. Despite widespread use in clinical practice for over 40 years, little is known about the systemic absorption and serum levels of corticosteroids following injection into the cervical epidural space. The literature surrounding the adverse effects of epidural corticosteroids has demonstrated adverse drug-drug interactions [2, 3], alterations in blood glucose levels among patients with diabetes [4, 5], and prolonged effects on the hypothalamic-pituitary-adrenal (HPA) axis [6]. These studies suggest that epidural corticosteroids have systemic pharmacologic effects, and the duration of action can be prolonged in subgroups of patients.

Recently, our group reported on the pharmacokinetics of lumbar epidural-administered triamcinolone [7]. The terminal elimination half-life of lumbar epidural-administered triamcinolone in a noncompartmental analysis was 523 hours, and the peak triamcinolone concentration of 4.1 ng/mL was detected within 24 hours after administration. This elimination half-life after lumbar epidural administration is much longer than the elimination half-life of intravenous administration and is likely explained by the suspension and redistribution of the depot preparation within the epidural fat and the epidural anatomy [7–9].

Knowledge of pharmacokinetic parameters of this corticosteroid following epidural administration into other areas of the spine beyond the lumbar region is necessary to help us better understand the duration of clinical effect and to compare the results to the results of lumbar epidural injection. Therefore, the primary objective of this study was to investigate the pharmacokinetics of fluoroscopy-guided cervical epidural-administered triamcinolone acetonide in a cohort of patients with cervical radicular pain seeking treatment at a pain medicine clinic.

## 2. Methods

This study was approved by the Mayo Foundation Institutional Review Board, and written informed consent was obtained from each patient prior to study participation. Although epidural administration of all corticosteroids including triamcinolone acetonide is considered “off-label” use and not approved by the US Food and Drug Administration, epidural administration of this medication occurs widely in clinical practice.

### 2.1. Study Participants

All patients were recruited from a pain medicine clinic at a tertiary referral medical center from 2012 through 2015. Patients referred for a C7-T1 interlaminar ESI were eligible for study recruitment. Inclusion criteria included (1) age older than 18 years (no upper age limit was specified), (2) cervical radicular symptoms, and (3) cervical spine magnetic resonance imaging within the past 24 months. Exclusion criteria included (1) cervical spine stenosis on magnetic resonance imaging; (2) use of exogenous steroid, including inhaled steroids, within the past 6 months; (3) history of any cervical spine surgery including fusion and decompressive laminectomy; (4) adrenal disease, pituitary disease, or other endocrinopathy known to impair the HPA axis; (5) end-stage liver (Child-Pugh classification A or greater) or kidney disease (creatinine clearance < 30 mL/min); (6) use of any medication known to interfere with the metabolism of triamcinolone acetonide, including, but not limited to, phenobarbital, phenytoin, itraconazole, rifampin, and selected antiretroviral agents; (7) presence of a psychiatric disorder that would limit obtaining informed consent or impair functioning in an ambulatory care environment (e.g., dementia, schizophrenia, and severe depression); and (8) pregnancy.

### 2.2. Fluoroscopy-Guided Cervical Epidural Steroid Injection

All fluoroscopically guided cervical interlaminar ESIs were performed in an ambulatory surgical center by a board-certified pain medicine physician. In the procedure suite, the patient was positioned prone on the fluoroscopy table, and the targeted skin area overlying the C7-T1 injection site was prepared with chlorhexidine antiseptic and sterilely draped. Following local anesthesia with 1% lidocaine, a 20-gauge Tuohy needle was introduced and advanced with intermittent fluoroscopic guidance to the level of the ligamentum flavum. The C7-T1 dorsal epidural space was entered using the loss-of-resistance to saline technique. Contrast injection (iohexol 300) in at least two views (lateral or contralateral oblique and anterior-posterior planes) was used to confirm the position of the needle in the epidural space and lack of intravascular or intrathecal uptake of the injectant. Following confirmation of the final needle position, triamcinolone acetonide 80 mg (triamcinolone acetonide injectable suspension (Kenalog) 40 mg/mL) in 2 mL of preservative-free normal saline was deposited in the epidural space. Following deposition of the corticosteroid, the needle was retracted from the epidural space, flushed with 0.5 mL preservative-free normal saline, restyletted, and removed.

### 2.3. Determination of Serum Triamcinolone Acetonide Levels

Serum triamcinolone acetonide levels were determined as previously described [10]. In brief, blood (5 mL) was collected by a trained phlebotomist prior to the ESI and on days 1, 2, 4, 6, 8, 14, 21, 28, 35, and 42 following the procedure. Triamcinolone acetonide was extracted from 0.5 mL of serum using an acetonitrile protein precipitation followed by methylene chloride liquid extraction of the supernatant. Seventeen microliters of the reconstituted sample extract was subjected to high-performance liquid chromatography and analyzed by tandem mass spectrometry. The calibration used a 5-point standard curve over a concentration range of 0 to 200 ng/mL.

### 2.4. Statistical Analysis

Triamcinolone serum concentration–time data were analyzed by noncompartmental methods using the software program WinNonlin (Certara, LP, St. Louis, Missouri). The apparent terminal elimination rate constants ([lambda]_z_) were determined by linear least-squares regression through 4 to 6 serum concentration–time points in the terminal elimination phase, as previously described [11]. The apparent elimination half-life (*t*_1/2_) was calculated as 0.693/[lambda]_z_.

## 3. Results

### 3.1. Baseline Demographics

The mean age of the study cohort was 47 years (range, 29–67 years). Eight patients, three males and five females, received a fluoroscopic guided C7-T1 ESI. There were no procedure-related complications.

### 3.2. Pharmacokinetics of Cervical Epidural-Administered Triamcinolone Acetonide

**Figure 1:**
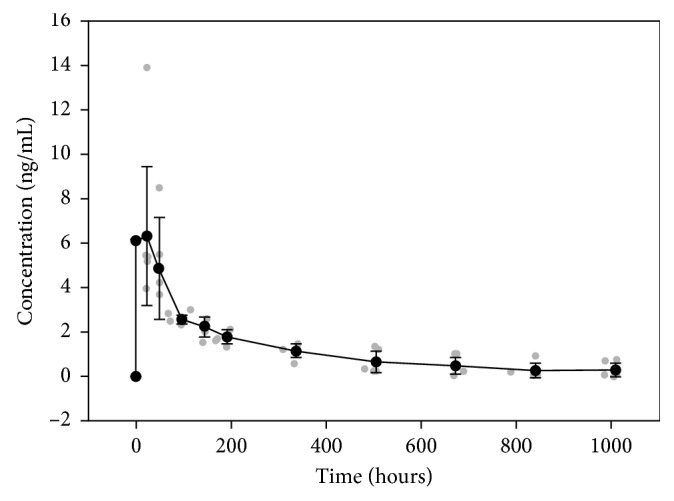
Graph of triamcinolone plasma concentration versus time following cervical epidural administration of triamcinolone acetonide. Individual data are represented by the grey circles. The black circles and error bars represent the mean and standard deviation, respectively.

Data for all 8 patients were fit by noncompartmental analysis. Peak triamcinolone concentrations (*C*_max_) of 5.4 ng/mL (median value) were detected within 22.1 hours (*T*_max_) of administration (Table 1). The terminal elimination half-life was 219 hours (median value). The graph in Figure 1 depicts the triamcinolone plasma concentration versus time following epidural administration of the drug.

## 4. Discussion

Recently our group reported the pharmacokinetics of lumbar epidural-administered triamcinolone [7]. For this current study, we sought to determine if there were similarities in cervical versus lumbar epidural triamcinolone administration. Therefore, the primary objective of this study was to investigate the pharmacokinetics of fluoroscopy-guided cervical epidural-administered triamcinolone acetonide in a cohort of patients with cervical radiculopathy seeking treatment at a pain medicine clinic. The main findings of this study were that the terminal elimination half-life of cervical epidural-administered triamcinolone acetonide in a noncompartmental analysis was 219 hours, and the peak triamcinolone concentration of 5.4 ng/mL was detected within 22.1 hours after administration.

The pharmacokinetics of epidural-administered triamcinolone acetonide are different compared to other routes of administration. Triamcinolone acetonide is a derivative of triamcinolone, and the suspension is commonly used for epidural injections [12–14]. Intravenous triamcinolone acetonide pharmacokinetics using the soluble form of this agent have been previously determined, demonstrating a half-life of approximately 1.5–2 hours [15, 16]. However, following intra-articular knee administration of the suspension, triamcinolone acetonide can be detected in serum for more than 2 weeks and the half-life ranges from 77 to 446 hours [17, 18]. Knowledge of pharmacokinetic parameters of this corticosteroid following epidural administration is necessary to better understand the duration of clinical effect and the potential risks and benefits of this medication as used in clinical practice.

**Figure 2:**
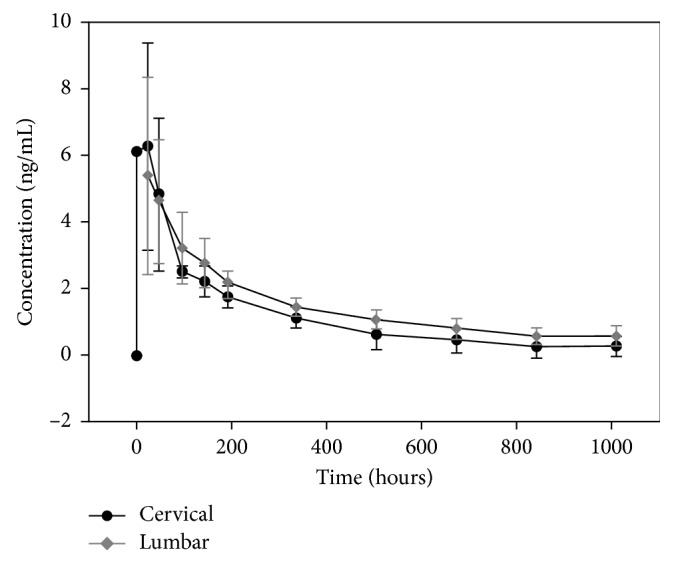
Graph of triamcinolone plasma concentration versus time comparing cervical versus lumbar epidural administration of triamcinolone acetonide. The black circles and gray diamonds represent the mean plasma levels of cervical and lumbar administration, respectively. While the pharmacokinetic profile is similar, the *T*_max_ is earlier and *T*_1/2_ is shorter for the cervical compared to the lumbar ESI. The lumbar data are from previously published data [7].

As discussed previously, our earlier study demonstrated that the elimination half-life after lumbar epidural administration is much longer than the elimination half-life of intravenous administration. This finding is most likely explained by the suspension and redistribution of the depot corticosteroid preparation within the epidural fat and the epidural anatomy [7–9]. Also, the injected volume may spread beyond the epidural space, epidural adipose tissue, and adipose located at the level of the foraminal canal which may serve as a site for reabsorption. There is adipose located within the dural cuff which may contribute as an additional site for drug reabsorption [19]. Epidural adipose tissue composition has similar distribution among all patients, considering the known variability between segments of the epidural space. We must also consider pathological conditions that may affect drug distribution and volume. In the current study, we found that the elimination half-life after cervical epidural administration is longer than after intravenous administration and that the pharmacokinetic behavior is similar to lumbar epidural steroid administration (Figure 2). However, the elimination half-life after cervical ESI is shorter than that seen after lumber ESI (219 versus 523 hours). The most likely possible explanations for this difference are anatomic differences. The cervical spinal canal cross-sectional area is smaller than the lumbar, and there is less epidural fat in the cervical epidural space [20, 21]. Accordingly, there is likely to be less depot steroid sequestration in the cervical epidural space, allowing for more rapid uptake within the cervical epidural vasculature. It is also known that that there are differences in the proportion of blood vessels to adipose tissue between the two areas with the cervical epidural space having relatively increased vascularity compared to the lumbar epidural space [20]. This would help to explain the relatively higher *C*_max_ (5.4 versus 4.1 ng/mL) and shorter half-life of cervical versus lumbar epidural triamcinolone.

Triamcinolone acetonide suspension is composed of crystalline particles ranging in size from 2.3 to 200 *µ*m [7, 9]. Following particle dissolution, this lipophilic corticosteroid then absorbs into epidural blood vessels and fat. Because distribution is primarily concentration driven, the dissolved drug is absorbed and distributed in blood, yielding a higher initial serum concentration. Over time, dissolution becomes rate limiting, and prolonged redistribution most likely occurs from epidural fat. Because the intravenous half-life is known, triamcinolone acetonide elimination from blood occurs at a greater rate compared with the movement of the drug from the epidural space. For these reasons, the elimination half-life that was calculated from the plasma concentration–time data represents an apparent half-life that results from first-order processes of dissolution of drug from the suspension, absorption of drug from the epidural injection site, and clearance of drug from the body. For intravenous injection of triamcinolone, clearance is determined by first-order elimination; for intra-articular administration, clearance is determined by first-order absorption and elimination; and for epidural administration, clearance is determined by first-order dissolution, absorption, and elimination. Thus, epidural administration of the suspension acts as a depot that slowly releases into the plasma over time, such that plasma concentrations are maintained for a much longer period compared with other routes of administration.

There are significant potential clinical implications of the prolonged *T*_1/2_ of epidural triamcinolone acetonide. It has been shown in other studies that exogenous administered corticosteroids may temporarily suppress the hypothalamic-pituitary-adrenal axis [22, 23]. This would be of potential significance, for example, if a patient underwent surgery or some other stressful exposure and was unable to mount a physiologic cortisol stress response. Similarly, exogenous corticosteroid administration has been associated with temporary blood glucose elevations and impaired blood glucose control in patients with diabetes [5, 6]. The longer *T*_1/2_ of epidural-administered triamcinolone carries with it, the possibility of prolonging the exposure to such adverse events.

This study has several limitations. First, epidural steroid disposition data were not available prior to this study. We anticipated that the *T*_1/2_ would be fairly long, and as a result of that assumption, 24 hours was chosen for the first data point. Given the calculated *T*_max_ of 22 hours for this study, in future studies, it would be prudent to obtain samples at earlier time points. Second, the pharmacokinetics from interlaminar cervical ESI may not be applicable to other techniques used to access the epidural space including a transforaminal or caudal approach. It is possible that variations in the anatomy of these regions of the epidural space could alter the observed pharmacokinetics. Third, the pharmacokinetics of triamcinolone should not be generalized to other commonly used epidural-administered corticosteroids, for example, methylprednisolone, betamethasone, or dexamethasone. Fourth, the observed pharmacokinetics may not be generalized to other groups of patients with lumbar spine pain including those with spinal stenosis, history of lumbar spine surgery, or other conditions that could alter the anatomy of the epidural space. Finally, age-related alterations in the dimensions of the epidural space have been well documented [24, 25]. These alterations could have influenced the spread of the injectate, which in turn could have led to differences in the concentration gradient of triamcinolone within the epidural space and subsequently affected the serum concentration of the drug. However, small age-related changes in the epidural space would more than likely have had a negligible effect on the study findings.

In conclusion, the peak serum concentration of triamcinolone following an interlaminar cervical ESI occurred at 22.1 hours, and the terminal elimination half-life was 219 hours. The pharmacokinetics of cervical epidural-administered triamcinolone is consistent with previously observed pharmacokinetics of lumbar epidural triamcinolone. The elimination half-life is considerably longer following epidural administration compared to intravenous administration, a finding most likely explained by the suspension and redistribution of the depot corticosteroid preparation within the epidural fat and the epidural anatomy. Future studies are needed to determine how the pharmacokinetics of thoracic interlaminar- and caudal-administered triamcinolone compares to lumbar- and cervical-administered triamcinolone and how the pharmacokinetics of other spinal corticosteroid injections such as zygapophyseal joint injections compares to epidural injection. In addition, the pharmacokinetics of other epidural-administered steroids should be determined and incorporated in clinical trials to investigate the potential associations between serum levels, clinical outcomes, and potential adverse endocrine effects.

## Figures and Tables

**Table 1:** Noncompartmental analysis of epidural-administered triamcinolone acetonide.

Subject	Half-life (h)	*T*_max_ (h)	*C*_max_ (ng/mL)
1	315	028	6.1
2	593	21.5	5.4
3	164	22.6	13.9
4	169	20.9	3.9
5	681	24.3	5.3
6	252	23.6	5.4
7	185	48.9	5.5
8	121	20.4	5.5
Average	310	22.8	6.4
SD	212	13.1	3.1
Median	219	22.1	5.4
Min	121	0.3	3.9
Max	681	48.9	13.9

*T*_max_ = time to maximum concentration; *C*_max_ = maximum.

## References

[1] Manchikanti L., Pampati V., Falco F. J., Hirsch J. A. (2013). Assessment of the growth of epidural injections in the medicare population from 2000 to 2011. *Pain Physician*.

[2] Hagan J. B., Erickson D., Singh R. J. (2010). Triamcinolone acetonide induced secondary adrenal insufficiency related to impaired CYP3A4 metabolism by coadministration of nefazodone. *Pain Medicine*.

[3] Song Y., Schroeder J. R., Bush L. M. (2013). Iatrogenicushing syndrome and secondary adrenal insufficiency related to concomitant triamcinolone and ritonavir administration: a case report and review. *Journal of the International Association of Providers of AIDS Care*.

[4] Even J. L., Crosby C. G., Song Y., McGirt M. J., Devin C. J. (1976). Effects of epidural steroid injections on blood glucose levels in patients with diabetes mellitus. *Spine*.

[5] Zufferey P., Bulliard C., Gremion G., Saugy M., So A. (2011). Systemic effects of epidural methylprednisolone injection on glucose tolerance in diabetic patients. *BMC Research Notes*.

[6] Elston M. S., Conaglen H. M., Hughes C., Meyer-Rochow G. Y., Conaglen J. V. (2013). Duration of cortisol suppression following a single dose of dexamethasone in healthy volunteers: a randomised double-blind placebo-controlled trial. *Anaesthesia and Intensive Care*.

[7] Hooten W. M., Nicholson W. T., Gazelka H. M., Reid J. M., Moeschler S. M., Lamer T. J. (2016). Serum triamcinolone levels following interlaminar epidural steroid injection. *Regional Anesthesia and Pain Medicine*.

[8] Yanez J. A., Remsberg C. M., Sayre C. L., Forrest M. L., Davies N. M. (2011). Flip-flop pharmacokinetics–delivering a reversal of disposition: challenges and opportunities during drug development. *Therapeutic Delivery*.

[9] Gazelka H. M., Burgher A. H., Huntoon M. A., Mantilla C. B., Hoelzer B. C. (2012). Determination of the particulate size and aggregation of clonidine and corticosteroids for epidural steroid injection. *Pain Physician*.

[10] Taylor R. L., Grebe S. K., Singh R. J. (2004). Quantitative, highly sensitive liquid chromatography-tandem mass spectrometry method for detection of synthetic corticosteroids. *Clinical Chemistry*.

[11] Gibaldi M., Perrier D. (1982). *Pharmacokinetics*.

[12] Ghahreman A., Ferch R., Bogduk N. (2010). The efficacy of transforaminal injection of steroids for the treatment of lumbar radicular pain. *Pain Medicine*.

[13] Cohen S. P., Bicket M. C., Jamison D., Wilkinson I., Rathmell J. P. (2013). Epidural steroids: a comprehensive, evidence-based review. *Regional Anesthesia and Pain Medicine*.

[14] Benyamin R. M., Manchikanti L., Parr A. T. (2012). The effectiveness of lumbar interlaminar epidural injections in managing chronic low back and lower extremity pain. *Pain Physician*.

[15] Derendorf H., Hochhaus G., Rohatagi S. (1995). Pharmacokinetics of triamcinolone acetonide after intravenous, oral, and inhaled administration. *Journal of Clinical Pharmacology*.

[16] Mollmann H., Rohdewald P., Schmidt E. W., Salomon V., Derendorf H. (1985). Pharmacokinetics of triamcinolone acetonide and its phosphate ester. *European Journal of Clinical Pharmacology*.

[17] Derendorf H., Mollmann H., Gruner A., Haack D., Gyselby G. (1986). Pharmacokinetics and pharmacodynamics of glucocorticoid suspensions after intra-articular administration. *Clinical Pharmacology and Therapeutics*.

[18] Beer P. M., Bakri S. J., Singh R. J., Liu W., Peters G. B., Miller M. (2003). Intraocular concentration and pharmacokinetics of triamcinolone acetonide after a single intravitreal injection. *Ophthalmology*.

[19] Reina M. A., Villanueva M. C., Maches F., Carrera A., López A., De Andrés J. A. (2008). The ultrastructure of the human spinal nerve root cuff in the lumbar spine. *Anesthesia & Analgesia*.

[20] Reina M. A., Franco C. D., Lopez A., De Andres J. A., van Zundert A. (2009). Clinical implications of epidural fat in the spinal canal. A scanning electron microscopic study. *Acta Anaesthesiologica Belgica*.

[21] Richardson J., Groen G. J. (2005). Applied epidural anatomy. *Continuing Education in Anaesthesia Critical Care & Pain*.

[22] Kay J., Findling J. W., Raff H. (1994). Epidural triamcinolone suppresses the pituitary-adrenal axis in human subjects. *Anesthesia & Analgesia*.

[23] Johnston P. C., Lansang M. C., Chatterjee S., Kennedy L. (2015). Intra-articular glucocorticoid injections and their effect on hypothalamic-pituitary-adrenal (HPA)-axis function. *Endocrine*.

[24] Hogan Q. H. (1996). Epidural anatomy examined by cryomicrotome section. Influence of age, vertebral level, and disease. *Regional Anesthesia*.

[25] Igarashi T., Hirabayashi Y., Shimizu R., Saitoh K., Fukuda H., Mitsuhata H. (1997). The lumbar extradural structure changes with increasing age. *British Journal of Anaesthesia*.

